# Load-Induced Changes of Inter-Limb Asymmetries in Dynamic Postural Control in Healthy Subjects

**DOI:** 10.3389/fnhum.2022.824730

**Published:** 2022-03-11

**Authors:** Jessica Heil

**Affiliations:** Institute of Sport Science, Carl von Ossietzky University Oldenburg, Oldenburg, Germany

**Keywords:** physical load, running, cycling, side-differences, injury risk, Y-Balance Test

## Abstract

Inter-limb asymmetries are associated with a higher potential risk for non-contact injuries. Differences in function or performance between the limbs might lead to imbalances and promote instability, increasing the potential risk for injuries. Consequently, an investigation of inter-limb asymmetries should be included in injury risk assessment. Furthermore, since non-contact injuries mainly occur under loaded conditions, an investigation of load-induced changes of inter-limb asymmetries can provide additional information on the athlete’s potential injury risk. Therefore, the current study aimed to investigate the influence of physical load on inter-limb asymmetries in dynamic postural control, which is essential in situations with a high risk for non-contact injuries such as landing, cutting, or stopping. In total, dynamic postural control of 128 active and healthy subjects (64 males and 64 females, age: 23.64 ± 2.44, height: 176.54 ± 8.96 cm, weight: 68.85 ± 10.98 kg) was examined. Dynamic postural control was tested with the Y-Balance Test (YBT) before and after a loading protocol on a bicycle ergometer or a treadmill. The results showed no significant increase of the inter-limb asymmetries in anterior direction [*F*_(1, 126)_ = 4.44, *p* = 0.04, η^2^*_*p*_* = 0.03]. Moreover, there is high variation between the subjects regarding the magnitude and the direction of the asymmetries and the changes due to load. Therefore, a more individual analysis considering the magnitude and the direction of the asymmetries is required. Thereby, considering different modifying factors, e.g., sex, injury history, and baseline level of asymmetries, can be helpful. Moreover, an analysis of the changes during load might provide further insights, reveal possible differences, and help detect the reasons and mechanisms underlying inter-limb asymmetries and asymmetrical loading.

## Introduction

Many athletes develop a difference in function or performance between their limbs, i.e., *inter-limb asymmetries* (Bishop et al., 2017). It might occur differences in strength, physical capacity, or balance (Bishop et al., 2018b; Dos’Santos et al., 2021; Helme et al., 2021). Such differences appear in different sports and might be a consequence of the sporting activity (Parrington and Ball, 2016). Many sports are mainly characterized by asymmetric (or unilateral) execution of movements with a preferred limb, such as kicking in soccer or throwing in handball (Bromley et al., 2021). Such unilateral movements possibly evoke inter-limb asymmetries (Parrington and Ball, 2016; Bishop et al., 2018b). However, inter-limb asymmetries occur not only in asymmetric but also in symmetric sports with mainly cyclic or alternating movement patterns, e.g., running, cycling, or swimming (Hart et al., 2016; Parrington and Ball, 2016; Maloney, 2019). These inter-limb asymmetries might be caused by the predominant use of one preferred limb, leading to differences in strength development, neural development, or uneven flexibility and range of motion in favor of the preferred limb (Parrington and Ball, 2016).

Inter-limb asymmetries are associated with a higher potential risk for non-contact injuries (Dos’Santos et al., 2021). They might lead to unequal force absorption or a loss of frontal plane stability which are essential to bear the impacting forces in situations with high unilateral loading and a higher risk for non-contact injuries, such as landing, cutting, or stopping (Paterno et al., 2010). Several studies investigated the relationship between certain side differences and occurring injuries (Helme et al., 2021). For example, asymmetries in movement competencies measured with a functional movement screen (FMS) (Chalmers et al., 2017; Attwood et al., 2019), Y-Balance Test (YBT) (Kiesel et al., 2014; Gonell et al., 2015), or Star Excursion Balance Test (SEBT) (Plisky et al., 2006) showed an association with non-contact injuries. However, the association of inter-limb asymmetries in dynamic force production tests, e.g., single-leg hop (SLH) (Brumitt et al., 2020) or in isolated muscle actions measured with isokinetic tests (Dauty et al., 2016), and injuries is not that clear due to an inconsistency of results (Helme et al., 2021; Guan et al., 2022). Nevertheless, additional consideration of side differences between the limbs can obtain further information about an athlete’s potential injury risk and should be implemented in injury risk assessment.

In this context, *dynamic postural control* plays an important role. Non-contact injuries mainly occur during dynamic actions, e.g., landing, cutting, or stopping. In these situations, the athletes must, among others, maintain stability in situations of high (unilateral) loading (Güler et al., 2020). Maintaining balance while the body is in motion is denoted as an athlete’s dynamic postural control (Johnston et al., 2018). Regarding the high number of dynamic actions in sports, the importance and necessity of good dynamic postural control in sports is not debatable (Whyte et al., 2015; Johnston et al., 2018). Moreover, poor dynamic postural control is associated with instability and reactive or compensatory movements. Instability and compensatory movements possibly increase the impacting load on the muscles, tendons, and ligaments and therewith the potential risk for lower limb injuries (Plisky et al., 2006; Wright et al., 2013; Whyte et al., 2015; Güler et al., 2020). Additionally, inter-limb asymmetries in dynamic postural control are also associated with a higher potential risk for lower limb injury (Helme et al., 2021). Relative and absolute side-differences measured with the YBT or SEBT, especially in the anterior reach direction, proved to be good precursors for sports injuries (Plisky et al., 2006; Stiffler et al., 2017; Helme et al., 2021). Therefore, it might help to assess inter-limb asymmetries in dynamic postural control to provide more insights into an athlete’s potential injury risk.

Furthermore, injuries typically occur during matches, competitions, or training when the athlete is physically stressed (Ekstrand et al., 2021). This might be ascribed to load-induced alterations of physiological processes, possibly leading to altered muscle patterns, reduced muscle activation, delayed muscle contraction, or decreased muscle-torque generation. These alterations possibly result in changes in risk factors, such as knee valgus, ground reaction forces, or dynamic postural control (Santamaria and Webster, 2010; Whyte et al., 2015; Barber-Westin and Noyes, 2017). Therefore, an analysis of the potential injury risk under loaded conditions and not only when the athlete is recovered is advisable, and analysis of inter-limb asymmetries under loaded conditions might gather additional insights into an athlete’s potential injury risk (Heil et al., 2020a; Verschueren et al., 2020).

Nevertheless, only a few studies have already examined the influence of physical load on inter-limb asymmetries (Heil et al., 2020a). Bromley et al. (2021), e.g., found a large effect of a soccer match on inter-limb asymmetries in eccentric impulses and peak forces during single-leg countermovement jumps, but only small and moderate effects in other parameters, e.g., peak landing force or peak landing impulse. Bell et al. (2016) showed no changes of inter-limb asymmetries in vertical ground reaction force after a standardized exercise protocol. Moreover, Bishop et al. (2021b) found a large effect of a repeated sprint protocol on jump height asymmetries. In contrast, Bromley et al. (2021) stated no significant changes in jump height asymmetries after a soccer match. About the influence of physical load on inter-limb asymmetries in dynamic postural control is not much known yet. Konstantopoulos et al. (2021) found an increase of inter-limb asymmetries in dynamic postural control measured with the YBT. Nevertheless, the protocol used in this study intended to induce local muscle stress on only one side of the body. Hence, still, nothing is known about more global protocols better reflecting the demands of sports and their influence on inter-limb asymmetries in dynamic postural control.

Overall, these findings indicate a possible change of inter-limb asymmetries due to load. Moreover, these findings also show a dependency of the results and changes on the measured parameter, the load type, the loading protocol, and the task used to measure a certain parameter. Consequently, to obtain reliable insights and deduce aspects for injury prevention practice, future studies need methods, i.e., load types, loading protocols, and tasks, reflecting the demands of the sporting context they should investigate. An athlete’s potential injury risk should be assessed under approximately real sporting conditions (Benjaminse et al., 2019; Bolt et al., 2021). In this context, since most sports require running, especially those with a higher risk for non-contact injuries, e.g., soccer, handball, or track and field sports, running protocols should be used. Nevertheless, many studies still use cycling protocols because they seem easier to apply in a laboratory setting (Johnston et al., 2018; Verschueren et al., 2021).

Therefore, the current study aimed to investigate load-induced changes of inter-limb asymmetries in dynamic postural in more representative conditions. Dynamic postural control was analyzed before and after two global loading protocols: a commonly used cycling protocol and a comparable running protocol. It was hypothesized that asymmetries in dynamic postural control would increase after physical load.

## Materials and Methods

The study was part of a bigger project considering the influence of physical load on dynamic postural control. The protocol was based on a study by Johnston et al. (2018) that was adapted and systematically replicated. The recorded data was analyzed in different studies considering different aspects (Heil et al., 2020b; Heil and Büsch, 2022). The whole project was conducted in accordance with the Declaration of Helsinki, and the local Ethics committee approved the protocol.

### Subjects

For the whole project *a priori* a sample size of *n* = 126 was determined with a power estimation (*F*-Test: η^2^*_*p*_* = 0.20, α = 0.01, 1-β = 0.99) for a multivariate three-way mixed analysis of variance (MANOVA) using G*power software (Vers. 3.1.9.7) (Faul et al., 2007). In total, 128 physically active and healthy people mainly normally trained (systematic training for 1–5 years) sport students (64 males and 64 females, age: 23.64 ± 2.44, height: 176.54 ± 8.96 cm, weight: 68.85 ± 10.98 kg) participated in the study (Table 1). The subjects were divided into two examination groups. One group completed the loading protocol on a cycle ergometer, and the other group completed the protocol on a treadmill.

**TABLE 1:** Subject characteristics.

	Total	Group 1	Group 2
		(Cycling)	(Running)
*N*	128 (64 m, 64 f)	64 (32 m, 32 f)	64 (32 m, 32 f)
Age (years) (*M* ± *SD*)	23.64 ± 2.44	24.11 ± 2.42	23.17 ± 2.37
Height (cm) (*M* ± *SD*)	176.54 ± 8.96	175.53 ± 8.17	177.56 ± 9.65
Weight (kg) (*M* ± *SD*)	68.85 ± 10.98	67.16 ± 10.08	70.51 ± 11.67
Leg length kicking leg (cm) (*M* ± *SD*)	96.09 ± 6.49	94.94 ± 6.54	97.24 ± 6.29
Leg length standing leg (cm) (*M* ± *SD*)	96.18 ± 6.53	94.94 ± 6.59	97.42 ± 6.28

*F, female; m, male; M, Mean, SD, standard deviation.*

To participate in the study, subjects had to fulfill different criteria: (1) No injuries in the lower limbs in the past 6 months. (2) Be able to perform the loading protocol. Ability was checked with the Physical Activity Readiness Questionnaire (PAR-Q) (Warburton et al., 2011). (3) No balance disorders or medication for balance disorders, no cardiovascular disease, no previous reports of chest pain, no neurological diseases, no vestibular or visual impairment, and no chronic ankle instability.

### Procedures

All subjects were tested in one 90-min session in a laboratory setting. At first, subjects were informed about the procedures and provided written informed consent to the experiment. Then, in a questionnaire, personal data, sporting background, injury history, laterality (kicking and standing leg), and the questions of the PAR-Q (Warburton et al., 2011) were prompted. Subjects not fulfilling eligibility criteria were excluded from the study.

Before testing, several anthropometric measurements (weight, height, leg length) were conducted. Weight was recorded using the InBody270 (InBody Co., Seoul, Korea), height was measured with a stadiometer (Seca GmbH & Co., KG, Hamburg, Germany), and measuring tape was used to measure leg length of both legs [distance between the subject’s anterior-superior iliac spine and the most distal part of the medial malleolus (Gribble and Hertel, 2003)]. Moreover, each subject performed four practice rounds of the YBT to get familiar with the testing procedures.

After a short resting period, testing procedures started. As pre-load measurement, three YBT rounds with 10 min rest in between were performed (20 min pre-load [pre01], 10 min pre-load [pre02], and immediately pre-load [pre03]). Then the subjects completed one of the two loading protocols. After the protocol, the subjects directly went back on the YBT for post-load measurement and had to perform one round of the YBT again.

### Instruments

#### Y-Balance Test

**FIGURE 1:**
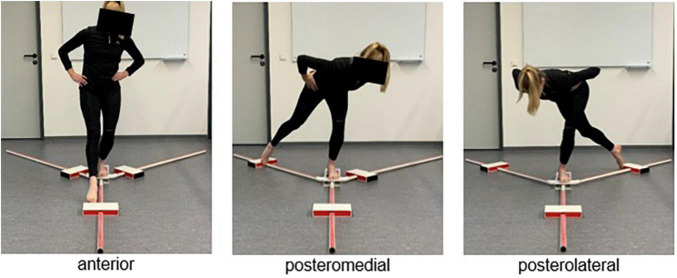
Y-Balance Test.

Dynamic postural control was measured with the YBT (Danville, VA, United States).^1^ For testing, the subject stands barefoot on a platform with one leg while sliding a block as far as possible in each direction of the YBT (anterior, posteromedial, posterolateral) with the other leg (Figure 1). During execution, the subject has the hands on the hips and tries to maintain balance. The reach distance (cm) in each direction is recorded. Dynamic postural control is investigated for the leg with whom the subject is standing on the platform. Trials were controlled according to the formerly published criteria by Plisky et al. (2009). If a trial was considered invalid, the subject must start over again with the current trial.

In the current study, one round of testing consisted of one trial on each leg. Meaning, after sliding the block in each direction on one leg, subjects returned to bilateral stance, switched sides, and conducted the YBT standing on the other leg. The starting leg varied between the subjects. In each group, one half of the subjects started the measurements with their preferred kicking leg, while the other half started with their preferred standing leg.

#### Loading Protocols

##### Cycling

As cycling protocol, a modified version of the Wingate Anaerobic-Test (Carey and Richardson, 2003) on a bicycle ergometer (Cyclus 2, RBM elektronik-automation GmbH, Leipzig, Germany) was conducted. Before the protocol started, each subject completed a 5-min warm-up (male: 90 RPM, female: 60 RPM). After a transition phase of 30 s (50–60 RPM), the protocol started, and subjects were instructed to accelerate and maintain their maximal effort for 60 s. Based on a former study from Johnston et al. (2018), the ergometer’s resistance was set at 7.5% of the subject’s weight. Heart rate was measured during the protocol with a Polar^®^ sensor.

##### Running

The running protocol was based on a protocol of Schnabel and Kindermann (1983) and performed on a treadmill (PPS 55med-I, WOODWAY GmbH, Weil am Rhein, Germany) with a slope of 7.5%. Before the protocol started, each subject completed a 5-min warm-up (8 km/h). Afterward, to determine the individual maximum velocity for the loading protocol, each subject had to complete an incremental test. The incremental protocol started with 8 km/h. Then the velocity was increased by 2 km/h every 20 s until the subject could not run at a certain speed. The final feasible speed was set as the subjects’ maximum velocity.

The running protocol started after a 5-min resting period. The treadmill was accelerated to the subject’s prior determined individual maximum velocity within 10 s and had a slope of 7.5%. The subjects had to run at this velocity until volitional exhaustion. This led to an average duration of about 60 s, which is comparable to the performed modified Wingate-Anaerobic-Test in cycling. Heart rate was measured with a Polar^®^ sensor during running.

### Inter-Limb Asymmetries

Inter-limb asymmetries were calculated with the following three steps:

1. The measured reach distances of the YBT were normalized to leg length using the following equation (Plisky et al., 2009):

(1) $$\begin{array}{c} \mathrm{Normalized}\mathrm{reach}\\ \mathrm{distance}\left(\mathrm{NRD}\right)\left(\%\right) \end{array}=\frac{\mathrm{reach}\mathrm{distance}\left(\mathrm{cm}\right)}{\mathrm{leg}\mathrm{length}\left(\mathrm{cm}\right)}\times 100$$

2. The three pre-load values of the kicking and standing leg were averaged:

(2) $$\begin{array}{c} \mathrm{Mean}\mathrm{normalized}\\ \mathrm{reach}\mathrm{distance}\left(\%\right) \end{array}=\frac{\begin{array}{c} \mathrm{NRD}\mathrm{pre01}+\mathrm{NRD}\mathrm{pre02}\\ +\mathrm{NRD}\mathrm{pre03} \end{array}}{3}$$

3. The percentage difference between the mean normalized reach distances was calculated according to Bishop et al. (2018a).

(3) $$\begin{array}{c} \text{Percentage}\\ \text{difference}\left(\text{PD}\right) \end{array}=\frac{100}{\max\text{value}}\times \min\text{value}\times \left(-1\right)+100$$

### Statistical Analysis

The data were analyzed using SPSS (version 28.0, IBM Corporation, Armonk, NY, United States). At first, the data were checked for normal distribution using the Shapiro-Wilk test. To determine the reliability of the normalized reach distances, the ICC (3,1) with an absolute agreement (Shrout and Fleiss, 1979) was used. ICC values were calculated across the three baseline measurements of the YBT and interpreted according to Koo and Li (2016) as > 0.9 = excellent, 0.75–0.9 = good, 0.5–0.75 = moderate, and < 0.5 = poor. Moreover, within-session reliability was assessed using the CV calculated as CV = [*SD* (trial pre01-pre03)/Mean (trial pre01-pre03)] × 100. CV values < 10% were acceptable according to Cormack et al. (2008). Additionally, to assess the degree of variation between the repeated measures for each leg, the standard error of measurement (SEM) was calculated as SD × √1-ICC.

With the given normalized reach distances, PD was calculated with the maximum and minimum values. Then, two-way mixed ANOVA (load type × time) was conducted to compare the PD between the two points of time (pre vs. post) and between the two load types (cycling vs. running). Significance level was set at *p* < 0.01.

Moreover, the direction of the asymmetries (kicking or standing leg) was determined with an IF Function in Microsoft Excel: *IF (kicking leg > standing leg, 1,2) (Bishop et al., 2021a). Kappa coefficients were calculated to determine the direction of the asymmetries and to analyze how consistently inter-limb asymmetries favored the same leg (kicking or standing leg) before and after the loading protocol. Kappa values were interpreted according to Viera and Garrett (2005), as 0.01–0.20 = slight, 0.21–0.40 = fair, 0.41–0.60 = moderate, 0.61–0.80 = substantial and 0.81–0.99 = almost perfect. Additionally, the subjects were divided into four direction types according to the direction of the asymmetries pre- and post-load: Group 1 (positive/positive), Group 2 (negative/negative), Group 3 (positive/negative), and Group 4 (negative/positive).

To include the direction of the asymmetries directly into the analysis, to display possible changes of the direction and to detect possible asymmetric loading between the legs, PD was also calculated between the kicking leg (kl) and the standing leg (sl).


(4) $$\begin{array}{c} \text{Percentage}\\ \text{difference}\left(\text{PD}\right) \end{array}=\frac{100}{\mathrm{value}\mathrm{kl}}\times \mathrm{value}\mathrm{sl}\times \left(-1\right)+100$$

Then, two-way mixed ANOVA (time × direction type) was conducted with the given PD values (kicking vs. standing leg) to compare the values between the four direction types pre- and post-load.

For all mixed ANOVAs, η^2^*_*p*_* is stated as effect size. Additionally, 90% CIs for the η^2^*_*p*_* were calculated using a SPSS syntax by Wuensch (2016). Moreover, effect sizes for repeated measures (Cohen’s d_*z*_) and 95% CIs for the adjacent points of time were also calculated using SPSS.

## Results

Regarding an athlete’s potential injury risk, only inter-limb asymmetries in the anterior (ANT) reach direction of the YBT are associated with a higher potential injury risk (Plisky et al., 2006; Stiffler et al., 2017; Helme et al., 2021). Therefore, only inter-limb asymmetries in ANT direction will be analyzed.

### Statistical Assumptions

The three baseline measurements in ANT direction showed excellent reliability and acceptable variability for the kicking (ICC = 0.96; CV = 2.85%; SEM = 0.34) and the standing leg (ICC = 0.96; CV = 2.66%, SEM = 0.34). According to West et al. (1995), all data were normally distributed due to a given skewness < 2 and kurtosis < 7. With the normalized reach distances, the PD was calculated and analyzed afterward. PD values are provided in Table 2. The raw data of the NRD and PD are provided in the Supplementary Material.

**TABLE 2:** Percentage difference anterior (max vs. min).

	Group		Pre	Post
PD ANT (%)	Total	*M* ± *SD*	4.29 ± 3.47	5.14 ± 4.73
		d [95% CI]		−0.19 [−0.36; −0.10]
		Average change		0.85 ± 4.57
		Change (%)		19.75
	Cycling	*M* ± *SD*	4.89 ± 4.15	6.35 ± 3.92
		d [95% CI]		−0.30 [−0.55; −0.05]
		Average change		1.46 ± 4.86
		Change (%)		29.79
	Running	*M* ± *SD*	3.69 ± 2.53	3.92 ± 4.30
		D [95% CI]		−0.06 [−0.30; 0.19]
		Average change		0.24 ± 4.21
		Change (%)		6.42

*ANT, anterior; CI, confidence interval; M, mean value; PD, percentage difference, SD, standard deviation.*

### Physical Load and Load Type

**FIGURE 2:**
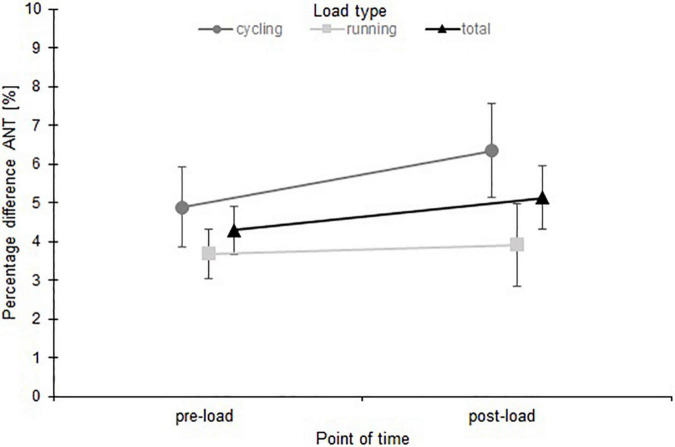
Mean values and 95% confidence intervals of the percentage difference anterior (max vs. min) for both load types (cycling and running) and for the total subject group.

A two-way mixed analysis of variance (time × load type) (Figure 2) showed no significant main effect of time [*F*_(1, 126)_ = 4.44, *p* = 0.04, η^2^*_*p*_* = 0.03] and no interaction between time and load type [*F*_(1, 126)_ = 3.31, *p* = 0.13, η^2^*_*p*_* = 0.03, 1-β = 0.33]. Between the subjects a significant difference was found between the two load types [*F*_(1, 126)_ = 9.41, *p* < 0.01, η^2^*_*p*_* = 0.07, 90% CI (0.02, 0.15)].

### Direction of Asymmetries

**FIGURE 3:**
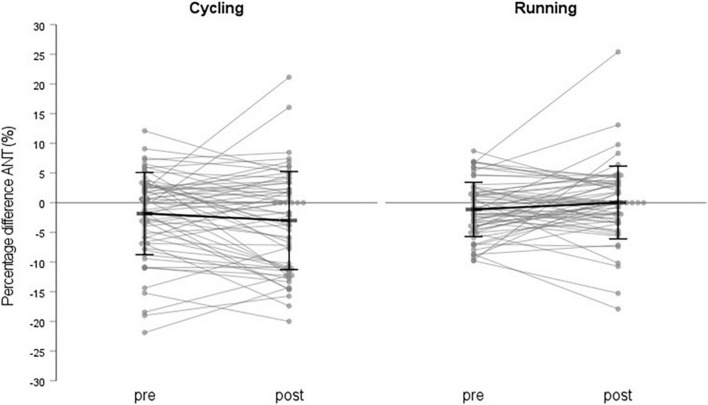
Mean values of percentage difference anterior (kicking vs. standing leg) pre- and post-load for the individual subjects separated between the two load types (cycling and running) and the mean values and standard deviation for the whole groups.

Regarding the direction of the asymmetries, the results show a moderate level of agreement for all subjects (Kappa = 0.41), moderate agreement for the cycling group (Kappa = 0.47), and a fair level of agreement for the running group (Kappa = 0.36). 90 of 128 subjects favored the same limb pre- and post-load (cycling = 47, running = 43), 38 did not. Figure 3 illustrates the PD values (kicking vs. standing leg) for the individual subjects pre- and post-load and was prepared using a SPSS syntax provided by Loffing (2022).

According to the direction of the asymmetries pre- and post-load the subjects were divided into four groups: Group 1 (positive/positive) *n* = 43, Group 2 (negative/negative) *n* = 47, Group 3 (positive/negative) *n* = 14 and Group 4 (negative/positive) *n* = 24. Table 3 shows the mean values for the four groups for the PD (kicking vs. standing leg) and the difference between pre and post.

**TABLE 3:** Mean values and standard deviation of the percentage difference anterior (kicking vs. standing leg) for the different direction types.

Direction type		PD (kl vs. sl)	PD (kl vs. sl)	Difference	Difference absolute
		Mean ± *SD* pre	Mean ± *SD* post	(post–pre)	(|post| —|pre|)
Pre	Post				
Positive*	Positive	3.79 ± 2.91	4.25 ± 5.07	0.46 ± 5.21	0.46 ± 5.21
Negative*	Negative	−6.37 ± 5.07	−7.82 ± 5.42	−1.45 ± 4.71	1.45 ± 4.71
Positive	Negative	2.36 ± 1.61	−5.56 ± 3.62	−7.92 ± 4.10	3.20 ± 3.82
Negative	Positive	−3.56 ± 2.54	3.02 ± 3.86	6.58 ± 4.25	−0.54 ± 4.97

*Positive, NRD kicking leg > NRD standing leg; Negative, NRD kicking leg < NRD standing leg.

*kl, kicking leg; NRD, normalized reach distance; PD, percentage difference; SD, standard deviation; sl, standing leg.*

**FIGURE 4:**
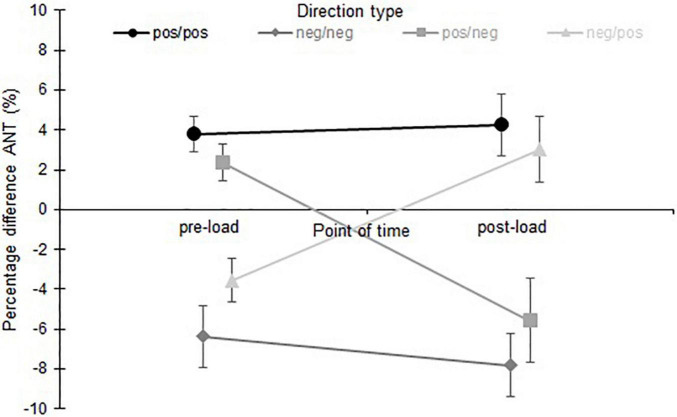
Mean values and 95% confidence intervals of percentage difference anterior (kicking leg vs. standing leg) pre- and post-load for the different direction types (positive/positive, negative/negative, positive/negative, negative/positive).

A comparison of the PD values (kicking vs. standing leg) pre- and post-load between the different direction types (Figure 4) with a two-way mixed ANOVA (time × direction type) showed no significant main effect of time [*F*_(1, 124)_ = 1.51, *p* = 0.22, η^2^*_*p*_* = 0.01, 1-β = 0.23] but a significant interaction between time and direction type [*F*_(3, 124)_ = 29.99, *p* < 0.001, η^2^*_*p*_* = 0.42, 90% CI (0.30, 0.50)]. Between the subjects a significant difference was found between the different direction types [*F*_(3, 124)_ = 71.28, *p* < 0.001, η^2^*_*p*_* = 0.63, 90% CI (0.54, 0.69)].

## Discussion

The current study aimed to investigate how inter-limb asymmetries in dynamic postural control change due to physical load. Dynamic postural control was chosen because it is essential in many sporting situations, e.g., cutting, landing, sprinting, or stopping (Güler et al., 2020). Moreover, deficits and inter-limb asymmetries in dynamic postural control are associated with a higher potential risk for lower limb injuries (Plisky et al., 2006; Stiffler et al., 2017; Helme et al., 2021). Therefore, an investigation of (inter-limb asymmetries in) dynamic postural control should be inevitable in injury prevention. Additionally, an investigation of risk factors and inter-limb asymmetries under loaded conditions can provide further information on an athlete’s potential injury risk since injuries mainly occur in loaded situations, e.g., during matches, competition, or training (Bourne et al., 2019; Verschueren et al., 2020). Recently, several studies investigated the influence of load on dynamic postural control (Wright et al., 2013; Johnston et al., 2018; Heil et al., 2020b). Nevertheless, up to now, only one study investigated the influence of a physical loading protocol on inter-limb asymmetries in dynamic postural control. Konstantopoulos et al. (2021) found increased inter-limb asymmetries after unilateral jumping. However, nothing is known about more global protocols better mimicking the real demands of sporting activities. Therefore, in the current study, inter-limb asymmetries of dynamic postural control were regarded and compared before and after physical load. It was hypothesized that inter-limb asymmetries would increase due to physical load.

This hypothesis could not be confirmed, and inter-limb asymmetries did not increase due to physical load. Moreover, two different types of load were compared because several studies regarding the influence of load on dynamic postural control and inter-limb asymmetries have shown that the changes seem to depend on the implemented load type and protocol (Wright et al., 2013; Heil et al., 2020a; Verschueren et al., 2020). Furthermore, studies often use cycling protocols (Johnston et al., 2018; Heil et al., 2020b; Verschueren et al., 2021), although running protocols seem to be the better choice because they are closer to the real sporting conditions. Therefore, a commonly used cycling protocol was compared to a comparable running protocol. Regarding the results, no differences were found between running and cycling. These findings indicate that the legs were possibly stressed equally due to both types of physical load, which seems not surprising concerning the symmetric/cyclic nature of the chosen loading protocols. However, it must be considered that the normalized reach distances were only decreasing after cycling but not after running (Heil and Büsch, 2022). Therefore, future studies should also take potentially upcoming differences in the internal load between different load types into account. In this context, protocols with longer durations, including changes in intensity and direction, should be used to better mimic the demands of sports, e.g., soccer or handball.

Although no increase in inter-limb asymmetries was found, nevertheless, there is a high variance (Figure 3). Moreover, an additional analysis of the direction of the asymmetries using kappa values showed only moderate agreement of the favored limb pre- and post-load. In 38 of the 128 subjects, the direction of the asymmetries changed after the loading protocol (Table 3). These results imply that the legs were maybe not equally stressed, although the magnitude of the inter-limb asymmetries was not significantly increasing. Therefore, a more individualized analysis of inter-limb asymmetries concerning the magnitude and direction and their changes due to physical load is indicated. Otherwise, different reactions of the legs might not be revealed and remain undetected only looking at the changes in the magnitude of inter-limb asymmetries.

In this context, it might be helpful to calculate the PD not only between the maximum and minimum value because this method cannot to show changes in the direction between different measurements and different points of time (Parkinson et al., 2021). It might be better to use one leg as a reference leg to evaluate changes in asymmetries and their direction due to training or physical load. Thereby, it is suggested to use a task-specific distinction or to use an inventory of questions or diagnostic tests to determine the preferred leg of an athlete (Dos’Santos et al., 2019; Virgile and Bishop, 2021). Moreover, it might also be beneficial to control/analyze the internal load of the legs during the loading protocol to detect possible differences and to detect asymmetrical loading and the mechanisms behind it. In this context, it could also help to mind possible modifying factors such as injury history, sporting background, or baseline level of asymmetries. Moreover, dynamic postural control is determined and influenced by other factors, e.g., anthropometric characteristics, sex, strength, or mobility (Fusco et al., 2020) and a consideration of these factors might help to detect the reasons for the changes of dynamic postural control and upcoming inter-limb asymmetries or asymmetrical loading.

Considering the given results in terms of injury prevention, the potential injury risk of the athletes is not increased due to higher inter-limb asymmetries. Nevertheless, a more detailed analysis and a consideration of the direction of the asymmetries showed that some subjects, however, possibly stressed their legs differently. Therefore, during injury risk assessment, not only a consideration of the changes in the magnitude of inter-limb asymmetries but also an observation of the changes in the direction of the asymmetries is indicated to reveal inter-limb asymmetries, respectively, asymmetric loading. Thereby, not only changes due to symmetric/cycling loading protocols should be concerned. Therefore, it might be helpful to use protocols with a longer duration, including changes of intensity and/or changes of direction, to create a design and a diagnostic that is more representative for the demands of sports with a higher risk for non-contact injuries (Whyte et al., 2015; Bolt et al., 2021).

Moreover, the current study had some more limitations that must be considered. (1) The current study used the YBT to measure dynamic postural control. Nevertheless, predicting the likelihood of injuries using the YBT has recently been doubted (Luedke et al., 2020). Moreover, the YBT preliminarily measures anticipative dynamic postural control, and in sports, mainly non-anticipative movements are present (Heil et al., 2020b). Therefore, it might be advantageous to use other balance tests besides the YBT to regard an athlete’s dynamic postural control. Additionally, a consideration of other elements, such as landing or cutting, could depict more aspects and risk factors occurring under real sporting conditions. (2) Moreover, it might be helpful to take different modifying factors into account. Thereby, a closer look might be taken at an athlete’s injury history. For example, it could help to record and concern the side of an athlete’s former injuries to distinguish between a healthy and an “injured” leg and to show if the legs still react differently to physical load after a certain period of rehabilitation. (3) Considering an athlete’s sporting background might help to detect if participating in a certain sport causes asymmetries and possible asymmetrical loading. Thereby it could be beneficial to differentiate between “symmetric” and “asymmetric” sports. Moreover, testing subjects from only one sport might reduce the number of potential sports conditional factors.

Altogether, no significant load-induced changes of inter-limb asymmetries in dynamic postural control were shown. Therefore, these findings indicate no increase in the potential injury risk due to higher inter-limb asymmetries. Nevertheless, assessing risk factors under physically loaded conditions seems advisable from a theoretical and practical perspective, especially when the high variation between the subjects is regarded. In this context, the current study shows several aspects that should be concerned during injury risk assessment. A consideration of possible modifying factors, such as injury history or sporting background, might provide further information. Moreover, further studies should regard changes in the magnitude and changes in the direction of inter-limb asymmetries to detect possible asymmetric loading. Furthermore, further studies using other protocols and tasks reflecting other aspects of sports are needed to confirm the current findings and to create a more comprehensive picture of an athlete’s potential injury risk and possible upcoming inter-limb asymmetries.

## Conclusion

The current study showed no significant changes of inter-limb asymmetries in dynamic postural control. This indicates that the limbs were possibly equally stressed due to physical load. However, an analysis of the direction of inter-limb asymmetries revealed possible asymmetric loading for some of the subjects. Therefore, it is suggested that changes in the magnitude and the direction of inter-limb asymmetries should be considered to obtain better insights into an athlete’s potential injury risk and avoid overlooking asymmetrical loading. Thereby a more individualized analysis of asymmetries and the consideration of possible modifying factors are indicated.

## Data Availability Statement

The original contributions presented in the study are included in the article/Supplementary Material, further inquiries can be directed to the corresponding author/s.

## Ethics Statement

The studies involving human participants were reviewed and approved by the Ethics Committee of the Carl von Ossietzky University of Oldenburg (EK/2020/035-02, 24 June 2020). The patients/participants provided their written informed consent to participate in this study.

## Author Contributions

The author confirms being the sole contributor of this work and has approved it for publication.

## Conflict of Interest

The author declares that the research was conducted in the absence of any commercial or financial relationships that could be construed as a potential conflict of interest.

## Publisher’s Note

All claims expressed in this article are solely those of the authors and do not necessarily represent those of their affiliated organizations, or those of the publisher, the editors and the reviewers. Any product that may be evaluated in this article, or claim that may be made by its manufacturer, is not guaranteed or endorsed by the publisher.

## Footnotes

1
functionalmovement.com
